# Supplementary material for: Retinal imaging with hand-held optical coherence tomography in older people with or without postoperative delirium after hip fracture surgery: A feasibility study
Source: PLoS One. 2024 Jul 16;19(7):e0305964. doi: 10.1371/journal.pone.0305964 (PMC11251583; doi:10.1371/journal.pone.0305964)
Supplement: Attachment — Submitted filename: Response to reviewers.docx [file pone.0305964.s002.docx]

Thank you for your review of our work

This is our response to the reviewer

Your comment

General comments:

The authors present a feasibility study on the assessment of the peripapillary nerve fiber layer (RNFL) thickness in patients experience postoperative delirium (DOL) after surgery for unilateral hip fracture. Despite a very small sample of 26 patients without and 4 patients with DOL the authors find significant differences in RNFL thickness between the two groups. While this certainly needs verification in a much larger sample, these results are very much of interest.

Our response

Thank you. We agree that a larger study is required to confirm or refute our findings

Major comments

The images were taken by one single operator who was in parts assisted by a colleague. Acquisition of OCT images using a handheld device can be very challenging for various reasons. It would have been very insightful if two people had been acquiring the images and their agreement was assessed. Same counts for retinal layer segmentation, especially if a custom tool for ImageJ was used to the assessments.

Our response

Indeed, it was challenging but actually quick to get used to

My training with the handheld device by the Leica engineer and also by our regional researchers who use handheld OCT was as a single user. Hence my approach of single operator albeit assisted in parts by a second operator.

Agreed, it would have been useful to have two people independently acquire images and compare their agreement. Unfortunately we didn’t. Perhaps in a future study.

Minor comments:
p.3 l.55:
Define “older”.

Our response

Thank you added

Minor comment

P.3. L.60:

Add a reference for POD hypothesis.

Our response

Thank you added

Your comment

P.4. l.88:

Thinning of the inner retinal layers (most notably RNFL and the IPL-GCL complex) has also been shown on macular OCT. Please include this and explain why it was chosen to assess the peripapillary RNFL. OCT acquisition of the macula is generally easier due to fixation.

Our response

Thank you.

Prior to this study we conducted a systematic review on OCT and MCI [Ref Noah]. The majority of studies our literature review identified were circumpapillary. Given that we are looking for potential preclinical changes that may evolve to cognitive impairment, we elected to remain circumpapillary in this study.

Your comment

P. l.88:

There is evidence of a thinning of the outer retinal layers in frontotemporal dementia. This should be included in the introduction.

Our response

Thank you we have added this.

Your comment

P.5 l.93:

Add references.

Our response

Thank you we have added this

Your comment

P.6 l.138:

Define “severe myopia”. Generally, <-6 diopters is used.

Our response

Thank you.

WE have clarified this in the manuscript in the manuscript

Your comment

P.8. L. 186:

I assume the age-adjusted normative values were taken from a measurements using a different OCT devise. This should be acknowledged and included in the limitations.

Our response

Thank you.

Your comments

P.9. L. 206:

Please define who the “OCT experts” were and how they were trained.

Our response

These are Ophthalmologists who conduct research using the handheld OCT device. They work out of the Ulverscroft eye Unit, University of Leicester and are co-authors in this work.

Comment

Table 2:

Please include lens status in the table.

Our response

Thank you for your comment. We acknowledge that some of our participants wear glasses. Unfortunately they could not remember what their refractive index was so we did not collect this data. This has been acknowledged as one of the limitations of the work.

Your comment

p.16 l.312:

Please stick to “cpRNFL” as the abbreviation.

Our response

Thank you.

Your comment

Figure 2:

The size of the segments varies. Please either unitize or explain in detail why this is the case.

Our response

Thank you

This is consistent across all the images we obtained from the software

A quick search for Copernicus OCT also shows images with this ‘asymmetry’

Thank you
